# Clinical pharmacy services in critical care: results of an observational study comparing ward-based with remote pharmacy services

**DOI:** 10.1007/s11096-023-01559-z

**Published:** 2023-04-08

**Authors:** Heike Hilgarth, Dominic Wichmann, Michael Baehr, Stefan Kluge, Claudia Langebrake

**Affiliations:** 1 Hospital Pharmacy, University Medical Centre Hamburg-Eppendorf, Hamburg, Germany; 2 Department of Intensive Care Medicine, University Medical Centre Hamburg-Eppendorf, Hamburg, Germany; 3 Department of Stem Cell Transplantation, University Medical Centre Hamburg-Eppendorf, Hamburg, Germany

**Keywords:** Critical care units, Clinical pharmacists, Hospitals, Pharmacist intervention, Telepharmacy

## Abstract

**Background:**

Pharmacists are essential team members in critical care and contribute to the safety of pharmacotherapy for this vulnerable group of patients, but little is known about remote pharmacy services in intensive care units (ICU).

**Aim:**

We compared the acceptance of pharmacist interventions (PI) in ICU patients working remotely with ward-based service. We evaluated both pharmacy services, including further information on PI, including reasons, actions and impact.

**Method:**

Over 5 months, a prospective single-centre observational study divided into two sequential phases (remote and ward-based) was performed on two ICU wards at a university hospital. After a structured medication review, PI identified were addressed to healthcare professionals. For documentation, the national database (ADKA-DokuPIK) was used. Acceptance was used as the primary endpoint. All data were analysed using descriptive methods.

**Results:**

In total, 605 PI resulted from 1023 medication reviews. Acceptance was 75% (228/304) for remote and 88% (265/301; *p* < 0.001) for ward-based services. Non-inferiority was not demonstrated. Most commonly, drug- (44% and 36%) and dose-related (36% and 35%) reasons were documented. Frequently, drugs were stopped/paused (31% and 29%) and dosage changed (31% and 30%). PI were classified as “error, no harm” (National Coordinating Council for Medication Error Reporting and Prevention [NCC MERP] categories B to D; 83% and 81%). The severity and clinical relevance were at least ranked as "significant" (68% and 66%) and at least as "important" for patients (77% and 83%).

**Conclusion:**

The way pharmacy services are provided influences the acceptance of PI. Remote pharmacy services may be seen as an addition, but acceptance rates in remote services failed to show non-inferiority.

**Supplementary Information:**

The online version contains supplementary material available at 10.1007/s11096-023-01559-z.

## Impact statements

- Pharmacist interventions are accepted less when provided by remote pharmacy services.
- Reasons, actions and impact of pharmacist interventions are comparable for ward-based and remote pharmacy services.
- Providing remote pharmacy services is less time consuming.
- In times of short resources, remote pharmacy services might be an option to offer essential pharmacy services (e.g., structured medication review).

## Introduction

Intensive care unit (ICU) patients are particularly vulnerable and at high risk for medication errors (ME) [1]. Furthermore, in ICU, a higher proportion of errors lead to patient harm than in non-ICU patients, often related to knowledge or performance failure [2, 3]. In general, ME can occur at any stage in the medication process and might prevent achieving therapeutic goals [4, 5].

In literature, “events or circumstances in drug therapy that actually or potentially interfere with the achievement of therapeutic goals” are referred to as drug-related problems, including ME, adverse events, and adverse drug reactions [5]. Pharmacists’ interventions (PI) employing “direct observation and evaluation of the patient and his/her medication-related needs; the initiation, modification, or discontinuation of patient-specific pharmacotherapy; and the ongoing pharmacotherapeutic monitoring and follow-up of patients in collaboration with other health professionals” are part of direct patient care [6]. For ICU, these activities reduce preventable adverse drug events and ME and have been shown to improve patient outcomes such as ICU length of stay and mortality [7–13].

The role of the critical care pharmacist is internationally defined and established, but this is different for Germany [14–17]. For ICU, the German Interdisciplinary Association of Critical Care and Emergency Medicine recommended the participation of a pharmacist on multi-disciplinary-team (MDT) rounds at least once a week and demanded the 24 h availability of a clinical pharmacist for consultation [18]. Furthermore, increasing digitalisation in the German health system offers new opportunities to provide pharmacy services and might support establishing pharmacists [19]. Additionally, remote pharmacy services, so-called “telepharmacy” could enhance existing services by extending pharmacists’ availability to 24 h per day and therefore providing services to complement day-time ward-based services in ICU [20, 21]. For many years, clinical pharmacists at the University Medical Centre Hamburg-Eppendorf (UKE) have been regularly providing direct patient service, including multi-disciplinary-team rounds, to the ICU with high acceptance rates of PI [22]. However, the steadily growing number of ICU patients and the limited number of clinical pharmacists require new concepts for pharmacy services.

### Aim

We compared the acceptance of pharmacist interventions (PI) in ICU patients working remotely with ward-based service. We evaluated both pharmacy services, including further information on PI, including reasons, actions and impact.

### Ethics approval

The study protocol was submitted to the ethics committee of the Hamburg Medical Association, but the need for approval was deemed unnecessary (23.02.2016, PV5226).

## Method

### Definitions

For this study we defined medication error (ME) as a “deviation from the optimal medication process, which leads or can lead to damage to the patient that is fundamentally avoidable” [5].

We defined drug-related problems (DRP) as "events or circumstances in drug therapy that actually or potentially prevent achieving desired therapeutic goals. DRPs include but are not limited to medication errors, adverse drug events, or adverse drug reactions" [5].

The term Pharmacist Intervention (PI) was defined as any action/communication (written or verbal) between pharmacists and healthcare professionals to modify drug use or optimise drug therapy. In addition, offering literature research as well as a proactive approach and discussion of pharmacotherapy and thereby contributing to avoiding solving DRP, as well as supporting the management of DRP, were included [23–25].

For the purpose of this study, a structured medication review was defined as “a structured evaluation of a patient’s medicines to optimise medicines uses and improving health outcomes. This entails detecting drug-related problems and recommending intervention” [26]. A “type 2b medication review” was performed using medication history and clinical data to evaluate indication-, drug- and monitoring-related issues [27].

A prescription of a drug with an Anatomical Therapeutic Chemical classification (ATC) according to the World Health Organisation, a blood product or component of enteral nutrition was counted as a valid medication line.

We referred to telepharmacy as “the provision of pharmacist care […] through the use of telecommunications or other technologies to patients […] at distances […]” [28].

### Study design

The prospective single-centre observational study was divided into two sequential phases involving two intensive care wards (interdisciplinary and surgical, 24 beds in total) at UKE. Direct patient care activities by pharmacists, e.g., MDT rounds, regular medication review, and continuous medication management, were already regularly provided. A comprehensive and structured medication review using the electronic health record (including all relevant patient data; ICM®-Dräger, Lübeck and Soarian®-Cerner, North Kansas City, Missouri, U.S) was performed once daily. Most patient cases will receive more than structured medication review during stay. In both phases, individual patient prescriptions were evaluated to identify and document possible DRP/PI. In addition, once daily, all relevant data were screened and collected as per study protocol during the structured medication review, but no data on comorbidities were collected. Medication-related data included the daily count of valid medication lines and their corresponding ATC-Codes. Patient-related clinical data included age (years), gender, renal function (as glomerular filtration rate [referred to as GFR in mL/min/1.73 m^2^] or renal replacement therapy), therapeutic intervention scoring system (TISS-10), simplified acute physiology score II (SAPS-II) and mode of ventilation (mechanical ventilation included both invasive and non-invasive ventilation with approved ventilators and required maintenance of a ventilation log). The electronic health record calculated SAPS-II and TISS-10 scores daily for patients with a stay of at least 24 h and no planned discharge that day. Patients were eligible for inclusion by being admitted to the two wards, aged at least 18 years and if they had a valid prescription. Patients who were admitted and discharged from the two wards or died before a medication review was conducted by the pharmacist were not included in the study population. For the ward-based phase, the pharmacist joined MDT ward rounds, was present on wards, and visited patients at the bedside to gain a more comprehensive overview of patients’ current health conditions.

The structured medication review was performed by a senior clinical pharmacist who was experienced (more than 13 years at ICU-wards; specialist pharmacist for clinical pharmacy and antibiotic stewardship expert) and a member of the MDT for more than 10 years. The communication of DRP/PI was either by telephone (21.06.–31.08.2017, remote phase) or in person (11.09.–22.11.2017, ward-based phase). The time needed to review patients and to provide ward-based or remote pharmacy service was recorded. The pharmacist’s working hours were from 9 am to 3 pm but not at weekends.

The acceptance of PI was used as a surrogate parameter for effectiveness and defined as a primary outcome. Using the categorisation of the ADKA-DokuPIK, there were six options of acceptance: Physician/nurse informed (1); PI proposed and implemented (2); PI proposed but not implemented (rejected) (3); PI proposed but not implemented (risk–benefit-assessment) (4); PI proposed, but outcome not known (5); problem not solved (6). The acceptance of PI was documented at the end of a ward-round or remote consultation or the next day. But as weekends were not covered, acceptance of PI on Fridays was checked the next working day, but at the latest, within 72 h. For this study, acceptance options 1 and 2 were described as “accepted”, and options 3–6 were regarded as “not accepted”. Finally, non-inferiority was defined after literature research revealed a ten percent-point absolute difference (mean) when comparing similar studies for ward-based (87% (CI 78.8–95.0)) and remote pharmacy services (78% (CI 76.5–78.5)) [7–9, 20, 21, 29, 30]. A 10-day wash-out period between phases was chosen to allow patients from the previous phase to be discharged. Additionally, intervention rates and time requirements for pharmacy services in both phases were analysed. For secondary outcomes, reasons, actions, impact as well as severity and clinical relevance were explored.

All PI were documented using a nationwide validated database (ADKA-DokuPIK), described previously [31, 32]. NCC-MERP-Index® (the National Coordinating Council for Medication Error Reporting and Prevention) was used for impact coding of PI also classified as ME [33, 34]. In addition, severity and clinical relevance were rated using an adapted score by Overhage and Lukes [35]. Both scores were independently evaluated by the senior pharmacist and two clinical experts (senior consultant and senior clinical pharmacist) to reduce bias. If mismatching (> 1 different category) occurred, a consensus between experts was sought. The Fleiss’ Kappa was used to calculate the inter-rater variability for both scores. For the interpretation agreement, the Kappa values (κ), according to Landis and Koch, were used [36].

### Statistical analysis

The study was powered to test non-inferiority of acceptance of PI by remote pharmacy services with a maximum deviation of 10 percentage points (absolute). Assuming a type I error of 5% and a power of 80%, the required number of PI for each phase was 296 PI. The software SPSS (Statistical Package for Social Science, IBM SPSS Statistics version 24, IBM Corp. New York) and Excel (Microsoft Office 365, version 1810) were used for analysis. The Chi-square-test (for categorical variables,) t-test (differences in mean) and the Mann–Whitney U-test (for variables that were not normally distributed) were used. *p* values of less than 0.05 were considered significant.

## Results

### Demographic and clinical characteristics

**Fig. 1:**
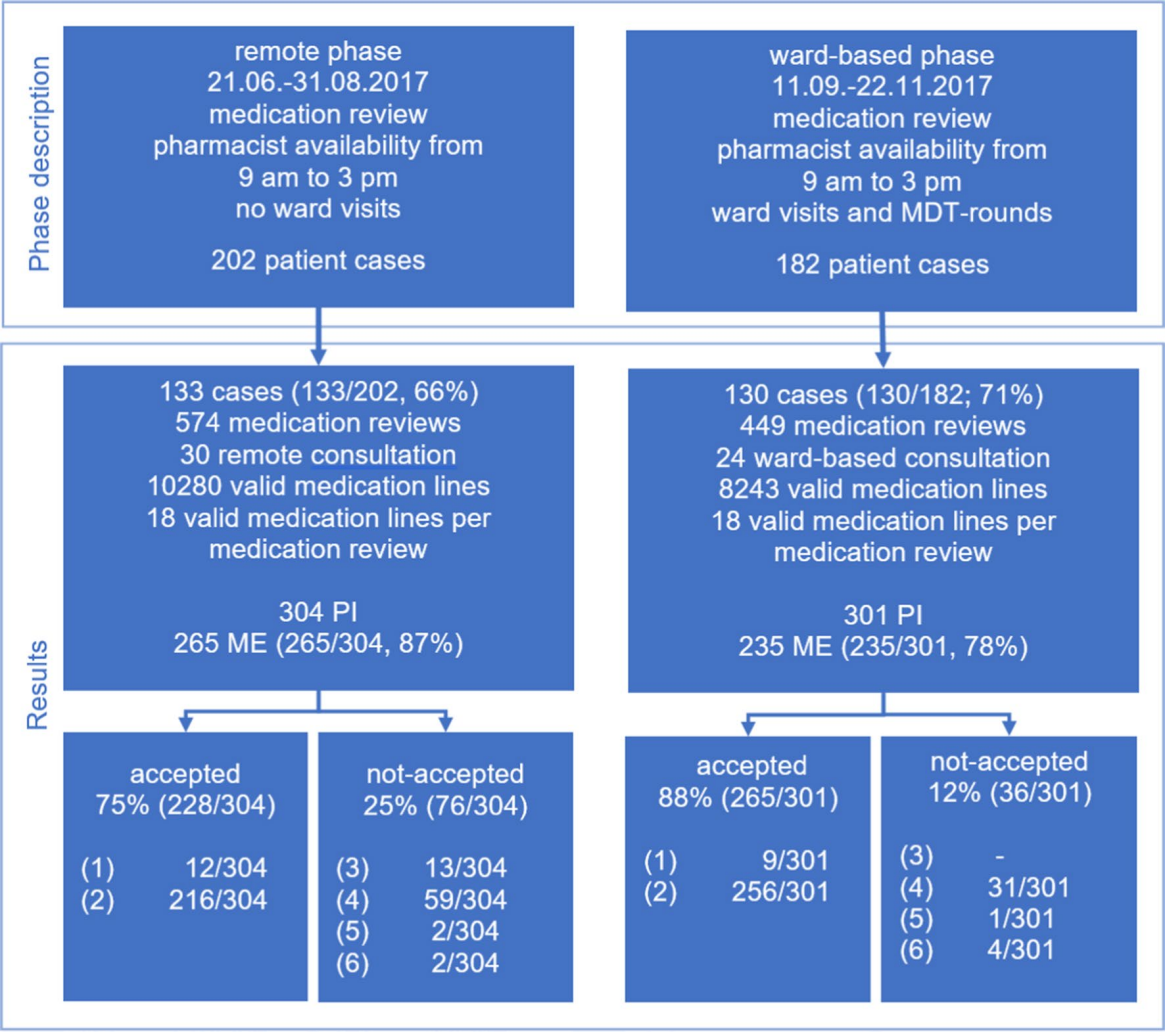
Study flow chart and description. PI, pharmacist interventions; CI, confidence interval; MDT, multi-disciplinary-team; ME, medication error; acceptance: physician/nurse informed (1); intervention proposed and implemented (2); intervention proposed but not implemented (proposal rejected) (3); intervention proposed but not implemented (risk–benefit-assessment) (4); intervention proposed, outcome not known (5); problem not solved (6)

We included 263 patient cases, corresponding to 133 (66%, 133/202, remote phase) and 130 (71%, 130/182, ward-based phase) patients, respectively. A total of 1023 structured medication reviews leading to 605 PI were documented (Fig. 1). For the remote phase (21.06.–31.08.2017), 574 structured medication reviews and 30 remote consultation sessions were recorded. In the ward-based phase (11.09.–22.11.2017), 449 structured medication reviews and 24 MDT ward rounds were documented. At least one PI resulted from a structured medication review in 36% (208/574) in the remote and 42% (188/449; *p* = 0.066; Chi-square-test). in the ward-based phase. Table 1 provides an overview of demographic patient data and details of medication reviews.

**Table 1:** Patient cases - demographics and information on structured medication reviews

	Remote phase	Ward-based phase	*p* value
**Intensive care patients (cases, n)**	**133**	**130**	
Age (years);mean (± standard deviation)	63 (± 17.1)	63 (± 13.2)	*p* = 0.709^a^
Male	64% (85/133)	63% (82/130)	*p* = 0.888^b^
Female	36% (48/133)	37% (48/130)	
Interdisciplinary ward	56% (75/133)	60% (77/130)	*p* = 0.641^b^
Surgical ward	44% (58/133)	40% (53/130)	
Medication reviews per patient case; (median, IQR)	2 (1.5–4)	2 (1–4)	
1 medication review per patient case	25% (33/133)	48% (63/130)	*p* < 0.001^b^
2 medication reviews per patient case	26% (35/133)	18% (23/130)	
3 medication reviews per patient case	19% (25/133)	8% (10/130)	
> 3 medication reviews per patient case	30% (40/133)	26% (34/130)	
Patients per remote consultation/MDT round; (median, IQR)	20 (19–22))	20 (19–21.75)	*p* = 0.958^a^
Pharmacist intervention per patient case (median, IQR)	1 (0–3)	1 (0–3.25)	*p* = 0.713^a^
**Medication reviews (n)**	**574**	**449**	
Medication review with pharmacist intervention	36% (208/574)	42% (188/449)	*p* = 0,066^b^
Pharmacist intervention per medication review, mean (± standard deviation)	0.53 (± 0.8)	0.67 (± 0.9)	*p* = 0.021^a^
Valid medication lines per medication review, (median, IQR)	18 (14–22)	19 (15–23)	*p* = 0.215^c^
Pharmacist intervention per remote consultation/MDT rounds; (median, IQR)	8 (6–14.25)	10.5 (8–15)	*p* = 0.177^a^
Mechanical ventilation	69% (396/574)	61% (273/449)	*p* = 0.888^b^
SAPS-II/TISS-10 information available^1^	540/574	427/449	
SAPS-II (median, IQR)	41 (33–50)	41 (31–49)	*p* = 0.401^c^
TISS-10 (median, IQR)	14 (10–17)	12 (8–17)	*p* < 0.001^a^
Renal function, information available (n)	98.9% (568/574)	99.8% (448/449)	
Renal replacement therapy (n)	31% (177/568)	45% (204/448)	*p* < 0.001^b^
GFR (mL/min/1.73m^2^, (median, IQR)	64 (40–93)	80.5 (48.25–103.75)	*p* < 0.001^c^

SAPS-II, simplified acute physiology score II; TISS-10, therapeutic intervention scoring system; MDT, multi-disciplinary-teams; IQR, interquartile range; GFR, glomerular filtration rate

^1^SAPS-II and TISS-10 data was not available, if patients had been onward for less than 24 h and were discharged that day

^a^Mann–Whitney-U-test; ^b^Chi-Square-test; ^c^t-test

Bold font is used to distinguish between data for patient cases and data which refers to medication reviews

### Primary outcome: acceptance of PI and intervention rates

The acceptance of PI was 75% (228/304, [CI 69.9–79.6]) in the remote and 88% (265/301, [CI 84–91.3], *p* < 0.001; Chi-square-test) in the ward-based phase. The distribution of non-acceptance is shown in Fig. 1. Acceptance and non-acceptance of PI varied for consultants (78% and 89%, *p* = 0.014; Chi-square-test), but not for junior (67% and 82%, *p* = 0.09; Chi-square-test) and senior doctors (80% and 90%, *p* = 0.094; Chi-square-test). In both phases, 1 PI per patient-case (remote phase IQR 0–3 and ward-based phase IQR 0–3.25) were documented, whereas PI per medication review (0.53 [± 0.8] and 0.67 [± 0.9], *p* = 0.021; Mann–Whitney-U-test) differed. The number of PI per remote consultation/MDT round (8 [IQR 6–14.25] and 10.5 [8–15], *p* = 0.177; Mann–Whitney-U-test) was comparable. The time requirements per PI (9 [IQR 7.3–16.8] and 18 [IQR 8.6–27.9] min; *p* < 0.001; Mann–Whitney-U-test) and per medication review (4 [IQR 3.9–5.4] and 12 [IQR 8.3–14.2] min; *p* = 0.009; Mann–Whitney-U-test) were significantly less in the remote phase. Accordingly, the total time requirements for a medication review and communication per day were less in the remote phase (92 [IQR 68–107.25] and 197 [IQR 128–250] min, *p* < 0.001; Mann–Whitney-U-test). The number of valid medication lines per review was similar in both phases (18 [IQR 14–22] and 19 [IQR 15–23]). The following drug groups were most often involved in PI: anti-infectives for systemic use (39% and 41%), blood and blood-forming organs (18% and 13%), nervous system (15% and 14%), alimentary tract and metabolism (10% and 7%) and cardiovascular system (7% and 9%).

### Secondary outcomes

For all PI (n = 605), 648 reasons were documented. Most PI had one (92% [281/304] and 93% [281/301]) and two (8% [23/304] and 7% [20/301]) reasons chosen. Drug- (44% [146/327] and 36% [118/312]) and dose-related reasons (36% [118/327] and 35% [111/321]) were most often. Other reasons (10% [32/327] and 18% [58/321]), such as drug counselling and assisting in drug choice, were less often (Supplement 1).

Overall, 83% (500/605) of PI were classified as ME, resulting in a significant difference between remote (87% [265/304]) and ward-based 78% [235/301] services (*p* = 0.004; Chi-square-test). Of these, 23% (62/265) in the remote and 10% (23/235) in the ward-based phase were not accepted. For ME, acceptance differed significantly (*p* < 0.001; Chi-square-test).

For all accepted PI (493/605), 561 actions were documented. The majority (87% each, [198/228] and [230/265]) of those had one action recorded. In 12% (27/228) and 13% (35/265), two actions were listed, and in one percent (remote phase, 3/228), three actions were recorded. Four of the five most documented actions coincided in the phases and varied only in order of precedence (Supplement 2).

**Fig. 2:**
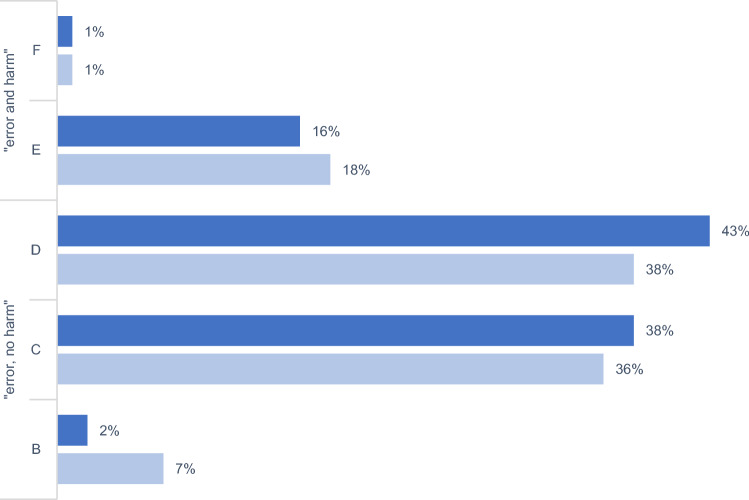
Impact of pharmacist intervention on patients according to NCC-MERP-Index® Evaluation, including all pharmacist interventions classified as ME (remote phase n = 265; ward-based phase n = 235). Remote phase = dark columns; ward-based phase = light columns. B an error occurred but the error did not reach the patient; C an error occurred that reached the patient but did not cause patient harm; D an error occurred that reached the patient and required monitoring to confirm that it resulted in no harm to the patient and/or required intervention to preclude harm; E an error occurred that may have contributed to or resulted in temporary harm to the patient and required intervention; F an error occurred that may have contributed to or resulted in temporary harm to the patient and required initial or prolonged hospitalization

**Fig. 3:**
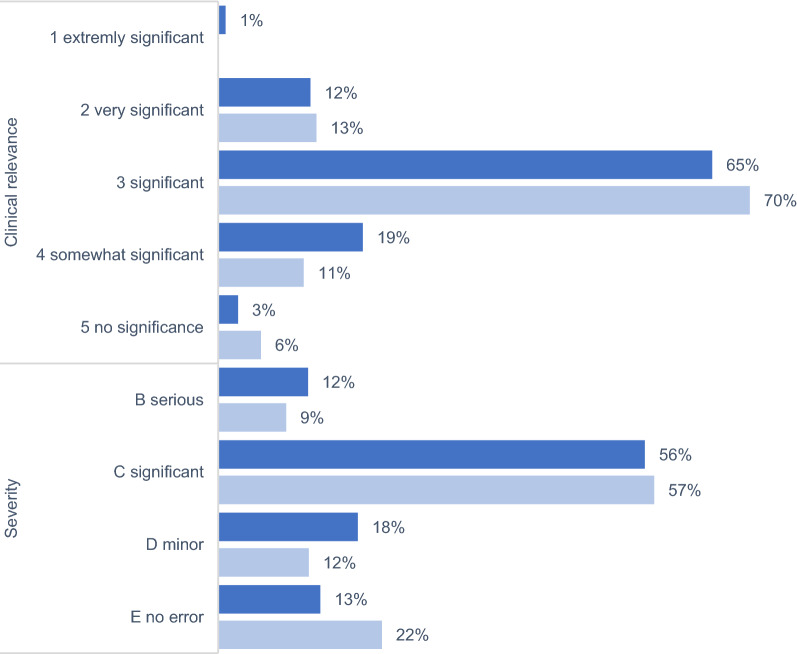
Severity and clinical relevance of pharmacist intervention adapted according [35], including all PI (remote phase n = 305; ward-based phase n = 301;). Remote phase = dark columns; ward-based phase = light columns

The NCC-MERP-Index® was used to evaluate the impact of PI, also classified as ME. The vast majority in both phases (83% [220/265] and 81% [191/235]) was classified as ‘error, no harm’ (categories B–D). Besides this, the proportion of ‘error and harm’ (categories E–F) accounted for 17% (45/265) and 19% (45/235), respectively (Fig. 2). For all PI (n = 605), an assessment of severity and clinical relevance revealed 68% (207/304) and 66% (200/301) as at least "significant" (category A–C). 78% (238/301) and 83% (250/301) were rated at least "important" (category 1–3) (Fig. 3). The distribution of NCC-MERP-categories was not different (*p* = 0.611; Chi-square-test), but the opposite was true for the assignment of severity and clinical relevance in both phases (*p* = 0.025; Chi-square-test). The interrater reliability Fleiss` Kappa was κ = 0.785 (substantial agreement) and κ = 0.88 (almost perfect agreement) for NCC-MERP-Index® evaluation and severity score and clinical relevance, respectively.

## Discussion

### Statement of key findings

The acceptance of PI was significantly different when working remotely compared to ward-based; therefore, the non-inferiority of remote working could not be shown. The way of providing pharmaceutical services has an important impact on acceptance.

### Interpretation

The acceptance of PI in both phases was similar to published literature [9, 20, 21, 29, 30, 37–39]. High rates (87.7–90%) were seen in hospitals with established ward-based pharmacy services, whereas lower rates (51–74%) were associated with newly implemented or remote-only services [8, 20, 21, 29, 37, 39–41]. But if remote pharmacists were used as a complementary measure/activity to an established ward-based pharmacy service, high acceptance rates were found (77–78%), which was also true for our study [20, 21]. Remote pharmacist activity or telepharmacy may offer an extension of pharmacy service to a 24 h/7 service and seems suitable to identify DRP and successfully address PI [20]. Full access to the electronic patient record (including laboratory and diagnostic data) and the already established ward-based pharmacy service could be responsible for high acceptance rates during both phases. The acceptance of PI was highest amongst consultants and senior doctors in the ward-based phase and might be attributed to communication skills and the presence at wards. Discussion and decisions about drug therapy are central aspects of MDT ward rounds, which consultants or senior doctors often lead. This might reflect the German health system's hierarchical structure, where junior doctors hesitate to accept interventions not being discussed with senior medical staff. But this may not apply to other settings or countries. At the same time, in the remote phase, there was a free choice of whom to contact and when. This hindered higher acceptance rates as decisions might have yet to be transferred to more senior staff or lost to follow-up by medical staff.

In both phases, 1 PI per patient case was documented, which is in line literature (remote: 1.7–2; ward-based: 0.5–4.6) [8, 21, 30, 40, 42–44]. The time requirements for a medication review and ward rounds pose a challenge for clinical pharmacists. For ward-based services, time requirements of 150–210 min are reported [8, 29, 42]. At least 180 min were needed to effectively review nine ICU patients and attend MDT rounds [8, 42]. In contrast, 5–20 min for checking and dispensing drugs were reported when pharmacists worked remotely [20]. Thus, time spent in the remote phase might have been less due to being independent of the ward routines (e.g., emergencies, admissions, or rounds), working without interruptions and not attending MDT rounds. Furthermore, patient cases with only one medication review were less in the remote phase, thus less time-consuming.

In both phases, the main reasons for PI were drug- and dose-related, and despite deviation in terminology, this is similar to the literature [8, 20, 21, 29, 37, 39, 45]. Actions resulting from an accepted PI were similar to those previously reported with recommendations to stop/pause drugs and dose-changes [21, 29, 30, 37–39, 46, 47]. There were no differences between the two phases in the evaluation of NCC-MERP-Index®. In line with literature, PI mainly prevent harm (“error no harm”, categories B–D) [8, 19, 39, 48]. This is attributed to the proactive nature of medication reviews and might indirectly show the impact on improving medication safety. Therefore, one can conclude that errors were common but usually remained without harm. Severity and clinical relevance assignments differ but are lower than previously described [37]. Independent of the classification as ME, the results highlight the role of clinical pharmacists in preventing harm.

### Strengths and weaknesses

A significant limitation is the single-centre and non-randomised study design. The selected sequential design could not be controlled for the severity of cases, seasonal fluctuations and the frequency of specific diagnoses. This contributed to a selection bias. Secondly, only one pharmacist who has been a member of the MDT for many years took part. Often several pharmacists would participate, supporting quality assurance and standardisation. On the other hand, regarding the limited availability of ‘clinical pharmacists’ in Germany, our study reflects reality [49]. To reduce bias, a panel of experts assessed the impact and severity and clinical relevance of PI. The high agreement values achieved for NCC-MERP-Index®, severity and clinical relevance confirmed the realistic assessment by the pharmacist. This may be due to the long-standing established pharmacy services to the wards and the professional experience of the rater. Thirdly, we did not differentiate between avoidable/unavoidable or potential errors. We focused on clinical pharmacists’ activities and acceptance of PI in ICU. Furthermore, not all technical means (e.g., video conferencing) were fully used in the remote phase, which might have influenced acceptance rates as well. Finally, offering remote pharmaceutical care might have been a new approach at the time of the study and could only be provided to a few ICUs in Germany.

Our study adds information about ward-based and remote pharmacy services to ICU. In addition, it is an example of the successful implementation of a clinical pharmacist as a member of the MDT.

### Further research

Telepharmacy is a valuable addition to standard pharmacy services. Locally—not only in the pandemic—we successfully used remote pharmacy service as an addition to/or replacement of ward-based services to ICU. Recently, pharmacists offered remote services for patients and hospital wards using telecommunication [40, 50]. Some have successfully adopted this concept and implemented telepharmaceutical care as part of tele-intensive care services [40]. In 2019, only 35% of ICUs in Germany had a pharmacy service implemented, most of them once weekly, and only 50% used electronic prescriptions [49]. In 2022 the updated “Recommendation on the structure and equipment of intensive care units” mentioned remote services/telepharmacy as an option to be provided in German ICUs. It stressed the impact of pharmacy services with direct patient-care activities [51]. As digitalisation moves forward, this might become a reasonable opportunity to provide pharmacy services more widely. It could also result in comparable acceptance rates as it becomes more common in daily practice. In light of further digitalisation, German hospitals might use remote pharmacy services or telepharmacy to enhance the quality and continuity of care, support ward-based activities, and contribute to further improvement of care.

## Conclusion

The study highlights that it is essential to consider how pharmacy services are provided to the wards. Despite ward-based approaches being more time-consuming than remote services, the acceptance of PI is higher, and so might be the impact on patient care. At the same time, it was shown that a pharmacist could identify many DRP/PI and ME working remotely or ward-based. Remote working and telepharmacy may be seen as an addition to standard ward-based services. But when used in this study, acceptance rates of PI failed to show non-inferiority compared to the ward-based approach. This should be an important fact to consider when planning to implement a pharmaceutical service in ICU, especially regarding costs and personnel [52].

## Supplementary Information

Below is the link to the electronic supplementary material.

Supplementary file1 (PNG 200 kb)

Supplementary file2 (PNG 103 kb)
