# Supplementary figures and images for: Clinical pharmacy services in critical care: results of an observational study comparing ward-based with remote pharmacy services
Source: Int J Clin Pharm. 2023 Apr 8;45(4):847–56. doi: 10.1007/s11096-023-01559-z (PMC10366025; doi:10.1007/s11096-023-01559-z)

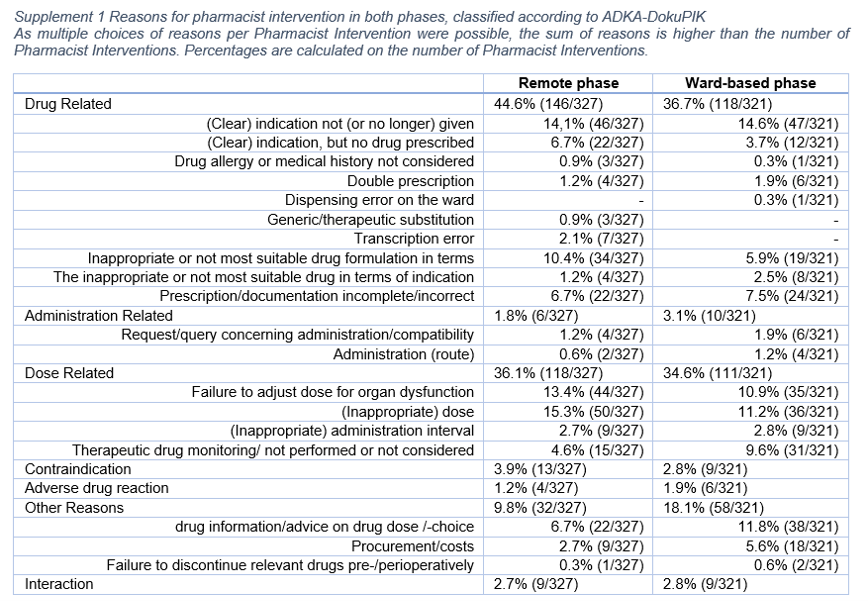

Supplement: Supplementary file 1 — Supplementary file1 (PNG 200 kb) [file 11096_2023_1559_MOESM1_ESM.png]

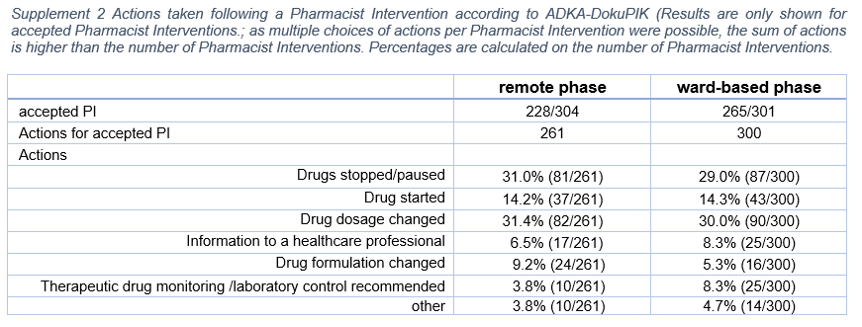

Supplement: Supplementary file 2 — Supplementary file2 (PNG 103 kb) [file 11096_2023_1559_MOESM2_ESM.png]
